# Cardiocirculatory and Metabolic Responses to Low- and High-Load Squat Exercise in Young and Middle-Aged Individuals

**DOI:** 10.3390/jfmk10030287

**Published:** 2025-07-25

**Authors:** Alessandro L. Colosio, Massimo Teso, Alberto Bottari, Luca Ferrari, Gianluca Bochicchio, Jan Boone, Silvia Pogliaghi

**Affiliations:** 1 Laboratoire Interuniversitaire de Biologie de la Motricité, Université Jean Monnet Saint-Etienne, 42023 Saint-Étienne, France; alessandro.colosio@univ-st-etienne.fr; 2 College of Health and Life Sciences, Hamad Bin Khalifa University, Doha P.O. Box 34110, Qatar; mteso@hbku.edu.qa; 3 Department of Neurosciences, Biomedicine and Movement Sciences, University of Verona, 37131 Verona, Italy; alberto.bottari@univr.it (A.B.); luca.ferrari_01@univr.it (L.F.); gianluca.bochicchio@univr.it (G.B.); 4 Department of Movement and Sports Sciences, Ghent University, 9000 Ghent, Belgium; jan.boone@ugent.be

**Keywords:** resistance training, strength training, ageing, exercise prescription, blood pressure, oxygen consumption

## Abstract

**Objectives**: The aim of this study was to assess the safety and feasibility of resistance training (RT) in middle-aged and young individuals by examining cardiocirculatory and metabolic responses to squat performed under low and high external loads as per current exercise prescription guidelines. **Methods**: Eighteen RT-trained individuals (nine middle-aged individuals, including eight women who were equally distributed) performed a cycling incremental test for the determination of their maximal aerobic capacity and three sessions of RT, respectively, to determine their one repetition maximum (1RM) of squat and their physiological responses during different training protocols of squat with equal training loads (3 × 12 at 55% 1RM vs. 5 × 5 at 80% 1RM). Whole-body metabolic (oxygen update and blood lactate) and cardiocirculatory (heart rate and blood pressure) responses and rate of perceived exertion (RPE) were compared across age groups and % 1RM to determine the metabolic stimulus and cardiovascular strain imposed by this form of training. **Results**: Young and middle-aged individuals exhibited similar cardiocirculatory responses to RT, with the only exception being a higher diastolic response in the middle-aged group for both protocols (present also at rest). No difference was found between the two age groups in terms of metabolic response and RPE. 80% 1RM induced a similar cardiocirculatory response and a higher RPE but a lower metabolic response compared to 55% 1RM. **Conclusions**: While no difference in physiological responses was found between the groups, the lower-load and higher-repetition training scheme demonstrated better time efficiency, metabolic activation, and perceived effort with equivalent cardiocirculatory strain. These findings support the safety of RT and can guide practitioners in the design of training protocols.

## 1. Introduction

Resistance training (RT) is a fundamental part of the exercise prescription for health over the whole lifespan, and its practice is recommended 2–3 days per week in order to ameliorate the size of muscles, strength, body composition, and health markers (e.g., blood pressure [1,2]. Combining elements of effectiveness and time efficiency, guidelines suggest the execution of exercises involving major muscle masses and external loads at around 60–70% of the one repetition maximum (1RM) in novices/intermediates and at ≥80% in experts [1]. These general landmarks should then be adapted depending on the situation, practical constraints, and safety considerations [3]. If, on the one hand, RT modalities are highly adaptable and manageable, they also introduce a certain level of complexity that necessitates supervision, particularly for novices engaging in RT [3]. Moreover, from a scientific perspective, this flexibility increases the number of challenges for the characterization of acute and chronic adaptations to RT, as it has been reported that even the first scientific guidelines were developed anecdotally, and scientists have only recently shifted toward a more physiological and evidence-based approach [4,5].

Due to the difference in muscle masses involved in RT exercises, the variety in exercise intensities, and the difficulty of applying the most common physiological measurements in RT settings, one aspect of this form of training that remains largely undescribed concerns cardiocirculatory and metabolic responses. In a holistic approach to RT prescription, knowledge of cardiocirculatory responses to RT allows for an informed evaluation of its risks [6], while awareness of the metabolic stimulus imposed by RT is necessary to individualize training prescription to specific objectives and evaluate the “concurrent benefit” of this form of training (e.g., the potential to mimic the improvements spurred by high-intensity interval training on peripheral and whole-body metabolism [7]).

From a cardiocirculatory perspective, constant practice of RT can ameliorate blood pressure profiles [8,9,10], but it could also entail a certain level of acute strain on the cardiovascular system, in turn limiting its applicability. Early studies on the acute response of blood pressure performed with invasive measurements during RT have reported values up to four times the resting blood pressure (mean pressure ~300 mmHg during exhaustive leg press [11]), surpassing the typical safety limits of around 220 mmHg of systolic pressure (PAs) during cardiopulmonary exercise testing [12]. However, recent investigations have reported more reassuring values, finding that both elderly people [13] and young participants [14] remain within the safety limit during RT, i.e., <200 mmHg. Still, these findings are restricted to relatively low-load RT (65% of 1RM) [13]) and young people [14], warranting further research in other populations with external loads that are at the higher end of the spectrum of current guidelines.

Regarding metabolic responses to RT, Lässing et al., 2023 have been the only researchers to provide concomitant measures of blood lactate ([La^−^]) accumulation and oxygen consumption ($\dot{V}$O_2_) during RT sessions performed with loads between 50 and 75% 1RM, i.e., measures to gain insight into the glycolytic and oxidative pathways of exercise [14]. However, these training sessions only involved young participants and were performed at a different % 1RM but with standard sets of 10 repetitions, while normally, in RT, a higher % 1RM corresponds to a smaller number of repetitions (and vice versa). This potentially hinders the direct translatability of these results to training practice. Moreover, Fernandes et al. (2018) reported that during a high-volume squat session, middle-aged adults experienced a comparable heart rate, rate of perceived exertion (RPE), and lactate level but significantly higher fatigue compared to younger individuals, despite identical external workloads [15]. Finally, Dello Iacono et al. (2020) reported, in older adults, a higher heart rate (HR) but a lower RPE and lower fatigue after manipulating the set structure using cluster sets, suggesting that the RT configuration may substantially influence acute strain and should be further investigated and taken into account when evaluating training safety and efficiency across age groups [16]. However, studies concurrently assessing cardiovascular, metabolic, and perceptual markers in this population remain scarce. In this context, middle age, as an intermediate stage between young and old, tends to present a declining fitness profile, including a lower maximal $\dot{V}$O_2_ ($\dot{V}$O_2_max), possible progressive deterioration of cardiovascular function, and a shift toward lower lean and higher fat mass [6]. Factors that, in turn, impact the maximum achievable external strength [17], active tissues, and circulatory responses to RT, thereby influencing training efficacy and safety. Furthermore, both the metabolic and cardiocirculatory responses to RT in the middle-aged population remain largely unexplored and warrant further investigation [1].

Based on the aforementioned considerations and the need for data characterizing the cardiocirculatory and metabolic responses to RT, the primary objective of this investigation was to bridge this research gap by examining the physiological responses in young and middle-aged individuals while training with external loads lying in the low and high ranges of exercise prescription guidelines. The squat exercise was selected due to its multi-joint nature, the engagement of large muscle groups, and its widespread utilization in RT practice. It was hypothesized that middle-aged individuals would exhibit comparable cardiocirculatory and metabolic responses to young healthy individuals, implying that RT would be equally feasible in both groups.

## 2. Materials and Methods

Eighteen subjects gave written informed consent to participate in the study (10 men, 8 women equally distributed between the young and middle-aged groups, Table 1). Inclusion criteria were age between 20 and 35 years for the young group and between 36 and 65 years for the middle-aged group, and a minimum of 6 months of RT experience; exclusion criteria were smoking and any cardiopulmonary or musculoskeletal condition or prescribed medication that could influence the physiological responses during testing. Experimental procedures were conducted at the University of Verona (University of Verona, Verona, Italy), approved by the Ethics Committee for Human Research from the University of Verona (CARP #54/2024), and conducted in conformity with the Declaration of Helsinki.

### 2.1. Protocol

On one preliminary occasion, the participants’ squat technique was checked by the same experienced researcher and certified strength and conditioning coach to ensure a consistent squat execution within the sample. Proper depth required to descend to a position with the thighs in parallel position with the ground, as defined by the trochanter head of the femur reaching the same horizontal plane as the superior border of the patella [18]. Then, after medical clearance, participants visited the laboratory on four occasions within a maximum of four weeks. On the first visit, the subjects performed a ramp incremental test to exhaustion for the determination of $\dot{V}$O_2max_ on an electromagnetically braked cycle ergometer (Sport Excalibur; Lode, Groningen, the Netherlands). The second visit consisted of the indirect determination of the maximum load that the subjects were able to lift during squat, and was considered as a participant 1RM. The last two sessions consisted of different training sessions of squat executed in random order. These training sessions were designed to have the same training load and different loading schemes, respectively, in the lower and higher ranges of the typical RT guidelines [6]: 55% of 1RM 3 sets × 12 repetitions (55% 1RM); 80% of 1RM 5 sets × 5 repetitions (80% 1RM). During these sessions, metabolic and cardiocirculatory responses were monitored as described below to characterize the acute physiological responses induced by two different training schemes. A period of at least seven days separated these two sessions to allow for full recovery. Participants were instructed to avoid caffeine consumption and physical activity, respectively, for at least 8 and 24 h before each testing session. Tests were conducted at the same time of the day in an environmentally controlled laboratory (22–25 °C, 55–65% relative humidity). Moreover, to minimize the variability of carbohydrate oxidation, participants consumed 2 h before all the testing sessions the same standardized meal, including 500 cc of water and 2 g of low glycaemic index carbohydrates per kg of body weight [19].

#### 2.1.1. Ramp Incremental Test

The ramp incremental test was performed on an electromagnetically braked cycle ergometer fitted to the individual preference of the participants (Sport Excalibur, Lode, Groningen, The Netherlands). The protocol utilized consisted of 4 min of baseline cycling at 20 W, followed by increases in power output (PO) based on the participant’s age: 25 W/min^−1^ for young and 15 W/min^−1^ for middle-age. Participants were asked to pick a self-selected cadence in the range of 70–90 rpm and to maintain it throughout the test. Failure to maintain the indicated cadence within 5 rpm (for longer than 5 s) during testing despite strong verbal encouragement was considered as the criterion for exhaustion. Breath-by-breath pulmonary gas exchange, ventilation, and heart rate were continuously measured using a portable metabolic cart (K5, Cosmed, Rome, Italy) as previously described [20].

#### 2.1.2. One Repetition Maximum Estimation

Upon arrival, participants started with a standard warm-up consisting of the following:

- 3 min of mobility exercises for the hips, back, and shoulders;
- 3 min of unloaded cycling;
- 2 min core activation consisting in the exercises of front plant, lateral plank, and back bridge;
- 2 sets of squat, respectively, 12 repetitions at 50% of the hypothetical 1RM, and 6 repetitions at 70% of the hypothetical 1RM. The hypothetical 1RM was the last self-reported 1RM of the participants. Between sets, a recovery of 4 min was given.

Then, subjects performed the maximal number of repetitions possible with a load corresponding to 80% of the hypothetical 1RM and the assistance of three spotters. The load lifted and the number of repetitions performed were then used to estimate 1RM according to the regression equation from Brzycki [21] and calculate the loads for the squat training sessions. Repetitions were considered valid when the thighs were in parallel position with the ground, as defined by the trochanter head of the femur reaching the same horizontal plane as the superior border of the patella [18]. This technical aspect was checked by the same experienced researcher and certified strength and conditioning specialist to ensure that proper form and depth were achieved. The same control was assured during the following testing sessions.

#### 2.1.3. Squat Training Sessions

To design sessions with almost equal training load, this was calculated in advance as follows: training load = % 1RM × total repetitions executed. A variety of set–repetition combinations (e.g., 50% × 2 × 12, 50% × 3 × 12, 50% × 4 × 12, 55 × %2 × 12, 55 × %3 × 12, etc.) were tested to identify protocols that imposed different external workloads (i.e., % 1RM), while yielding a similar total training load. [22]. Of these combinations, the two providing the best results were the following:

- 3 sets × 12 repetitions at 55% 1RM (training load of 1980 arbitrary units);
- 5 sets × 5 repetitions at 80% 1RM (training load of 2000 arbitrary units).

This approach allowed us to match training loads across protocols while maintaining set and repetition schemes that are commonly used in practical RT contexts. However, this also meant that set duration and time under tension were not standardized between conditions, since the number of repetitions per set differed. Tests were performed in a randomized order. Each squat session was preceded by the same warm-up presented for 1RM determination, but with slightly lower intensity for the 2 warm-up sets of squat in order to minimize metabolic activation (warm-up set 1: 12 repetitions at 25% of 1RM, set 2: 6 repetitions at 45% of 1RM), with 3 min and 30 s of recovery in between. Between the core activation and squat warm-up sets, subjects were also equipped with the portable metabolic cart and rested for 5 min in sitting position to record baseline values. Upon the start of the training protocol, the same recovery in sitting position was maintained between each set. The start and end of the sets were considered as the moments at which the barbell was lifted from or repositionedon the squat ruck, respectively.

**Figure 1:**
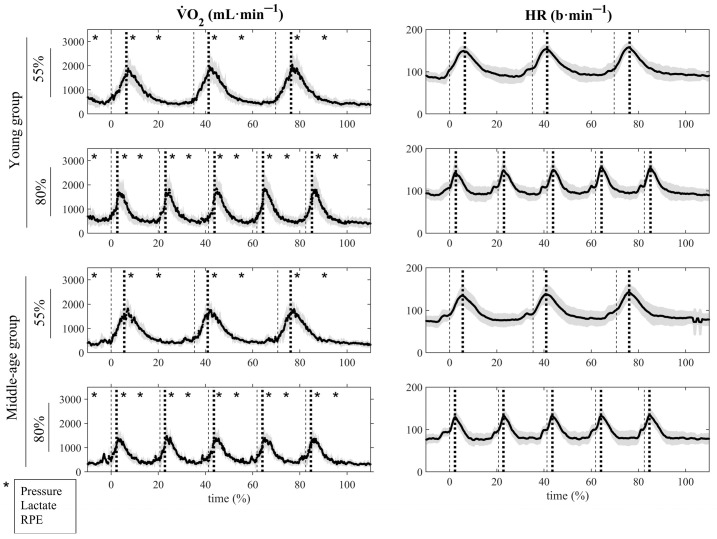
Mean (●) ± SD (grey areas) values of $\dot{V}$O_2_ and HR are plotted over time, expressed as a percentage (%), during the 55% 1RM and 80% 1RM resistance training protocols, in young (top panels) and middle-aged (bottom panels) individuals. The dashed line (---) and dotted line (∙∙∙) represent the start and end of each series, respectively. * Indicates the punctual times of blood pressure, lactate, and RPE data collection.

During the protocol, pulmonary gas exchange and heart rate were continuously measured using the same metabolic cart described for the ramp incremental test. Capillary blood samples (20 μL) were drawn from the earlobe at baseline, immediately after each set and at 1 min 30 s of recovery. The samples were immediately analyzed to measure blood lactate concentration (Biosen C-Line, EKF Diagnostics, Barleben, Germany). Moreover, at the same time as blood lactate, blood pressure was measured using a semiautomatic blood pressure monitor (Suntech Tango+, SunTech Medical, Morrisville, NC, USA), and the participants were asked to report their perceptual response to exercise using a 0–100 RPE scale [23]. A schematic representation of this procedure is depicted in Figure 1.

### 2.2. Data Analysis

#### 2.2.1. Ramp Incremental Test

For the gas exchange variables, aberrant data points that lay 3 SD from the local mean were removed, and trials were linearly interpolated on a 1 s basis, and then averaged every 5 s. $\dot{V}$O_2max_ was determined as the highest $\dot{V}$O_2_ obtained over a 10 s interval [20].

#### 2.2.2. Squat Training Sessions

Gas exchange variables were treated with the same procedure used for the ramp incremental test. In addition, data were visually inspected to detect possible outliers in the respiratory data exceeding $\dot{V}$O_2max_ in the transition between standing to sitting position and, therefore, considered not physiological.

For the $\dot{V}$O_2_, ventilation ($\dot{V}$E), respiratory frequency (Rf), and HR variables, the baseline value was calculated as the average of the last 30 s preceding the two sets of squat warm-up. During the training protocol, the analysis window for respiratory variables extended from the start of each set to 90 s after its completion. This was chosen to account for the known temporal delay in $\dot{V}$O_2_ kinetics relative to muscular energy demand [24]. The peak value was defined as the highest measurement obtained at either time point. $\dot{V}$O_2_ and HR were also expressed as % values relative to the recorded maximum $\dot{V}$O_2_ (i.e., $\dot{V}$O_2max_) and HR (i.e., highest value recorded) during the incremental test.

Baseline [La^−^], RPE, and systolic and diastolic (PAd) blood pressure values were collected during the 5 min before the squat warm-up sets, while the peak responses were considered the as the highest values recorded immediately after the sets for RPE and systolic and diastolic blood pressure, and as the highest value recorded in the whole recovery (immediately after or at 1.5 min) for [La^−^]. This approach was chosen to account for the different dynamics of circulatory variables and the time required for [La^−^] to appear in the bloodstream [25]. Double product (DP) values were calculated for a given time point as the product between systolic blood pressure and heart rate.

Finally, a session-representative value for all variables was obtained by averaging the peak values recorded after each set.

Signal analysis was performed by MatLab (Version R2021B, MathWorks Inc, Natick, MA, USA) scripts, and statistical analysis was performed using Sigmaplot (Version 12, Systat Software Inc., San Jose, CA, USA).

### 2.3. Statistics

Data are presented as mean ± standard deviation (SD). Differences between the young and middle-aged groups in age, anthropometrics, metabolic, and cardiovascular and muscle strength characteristics were investigated using *t*-tests. After assumption verification (i.e., normality, homogeneity of variance), two-way repeated measure analysis of variance (RM ANOVA, protocol × age) was used to assess differences in the metabolic ($\dot{V}$O_2_ $\dot{V}$O_2_, $\dot{V}$E, Rf, [La^−^], and % $\dot{V}$O_2_), cardiovascular (PAs, PAd, HR, DP, and % HR), and RPE variables in response to the two different training protocols in the young and middle-aged groups. Post-hoc analyses were performed using the Holm–Sidak method. The sample size was determined based on a power analysis using G*Power (version 3.1). For the primary outcome variable $\dot{V}$O_2_, with an expected medium effect size (f = 0.5), an alpha level of 0.05, and a power of 0.80, the required sample size was calculated as 12 participants. Although the effect size for the analysis was set at 0.5, the actual effect size, based on the difference in $\dot{V}$O_2max_ between young and middle-aged individuals [26] would have been substantially larger. The final sample of 18 participants exceeded the minimum required size and provided additional power to detect differences, especially on other variables. α was set in advance at the 0.05 level. Statistical significance was accepted when *p* < α.

## 3. Results

Participants’ characteristics are presented in Table 1. Young and middle-aged groups differed in terms of age (25.6 ± 2.2 vs. 50.1 ± 5.4 years, *p* ≤ 0.001), normalized $\dot{V}$O_2max_ (42.7 ± 6.6 vs. 34.0 ± 6.4 mL·kg^−1^·min^−1^, *p* = 0.012), maximal HR (192.3 ± 4.4 vs. 176.6 ± 7.4 b·min^−1^, *p* ≤ 0.001), baseline PAd (67.8 ± 6.8 vs. 90.5 ± 7.0 mmHg, *p* ≤ 0.001), and normalized strength (1.3 ± 0.2 vs. 1.0 ± 0.3 kg·kg body weight, *p* = 0.028), while no difference was present for the anthropometrical characteristics, PAs, and absolute 1RM of squat (Table 1).

**Figure 2:**
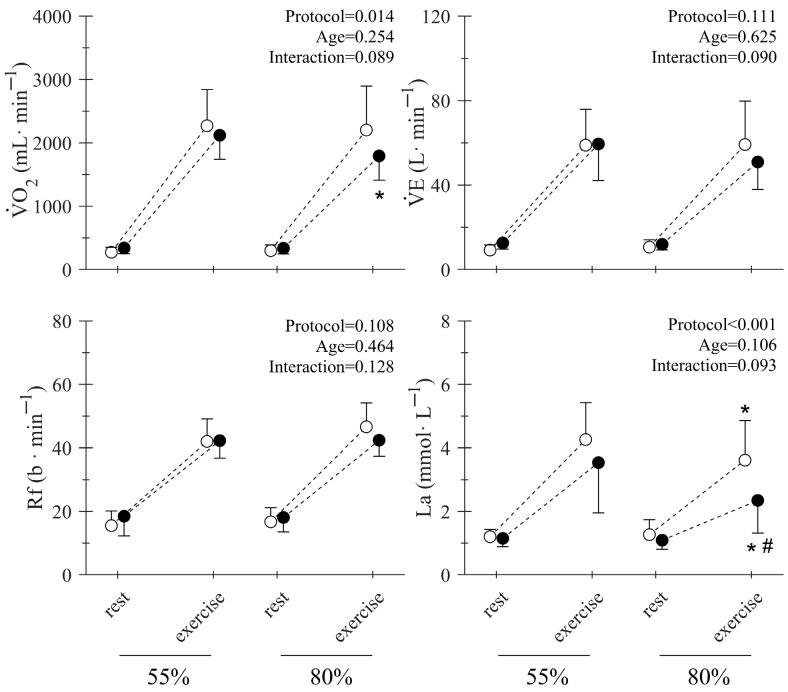
Rest and mean peak responses during the 55% 1RM (circles) and 80% 1RM (squares) squat (exercise) for oxygen uptake ($\dot{V}$O_2_), ventilation ($\dot{V}$E), respiratory frequency (Rf), and blood lactate concentration (La) in young (white) and middle-aged (black) subjects. The main effects of the 2-way RM ANOVAs (protocol × age) are displayed in the upper right corner of each panel. * Indicates a significant difference with 55% 1RM. # Indicates a significant difference with the young group.

Regarding the cardiocirculatory responses to squat training, the only detected main effect by ANOVA was the effect of age on the PAd and HR response (Figure 2, main effect of age on PAd: *p* < 0.001, HR: *p* = 0.031). Post hoc analysis detected statistical differences between the PAd of the young and middle-aged participants both at baseline and during exercise (Figure 2) for the 55% 1RM and 80% 1RM protocols, while HR in these two groups was different only during the exercise phase of the 80% 1RM protocol.

**Figure 3:**
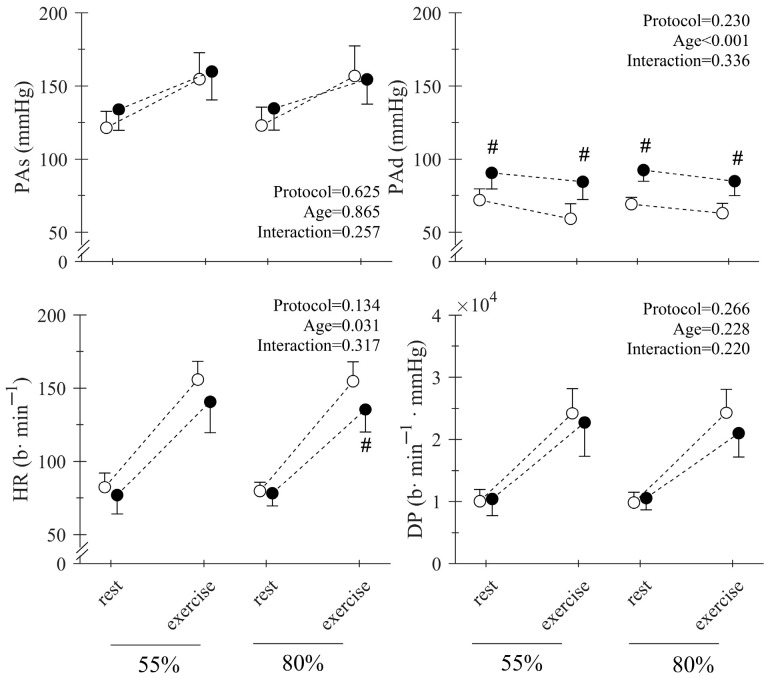
Rest and mean peak responses during the 55%1RM (circles) and 80%1RM (squares) squat (exercise) for systolic pressure (PAs), diastolic pressure (PAd), heart rate (HR), and double product (DP) in young (white) and middle-aged (black) subjects. The main effects of the 2-way RM ANOVAs (protocol × age) are displayed in the upper right corner of each panel. # Indicates a significant difference with the young group.

No significant differences were detected in the metabolic response to the 55% 1RM and 80% 1RM training protocols between young and middle-aged individuals, except a tendency for lower blood lactate accumulation in the middle-aged group during the 80% 1RM condition (main effect of age on [La^−^]: *p* = 0.106; Figure 2). ANOVA also revealed a main effect of the protocol investigated on the $\dot{V}$O_2_ and [La^−^] responses, but not in $\dot{V}$E and Rf (Figure 3 main effect of protocol on $\dot{V}$O_2_: *p* = 0.014, [La^−^] *p* < 0.001). Post hoc analysis showed a difference in the $\dot{V}$O_2_ response of middle-aged participants between 55% 1RM and 80% 1RM and a difference in the [La^−^] of both young and middle-aged participants between 55% 1RM and 80% 1RM (Figure 3).

**Figure 4:**
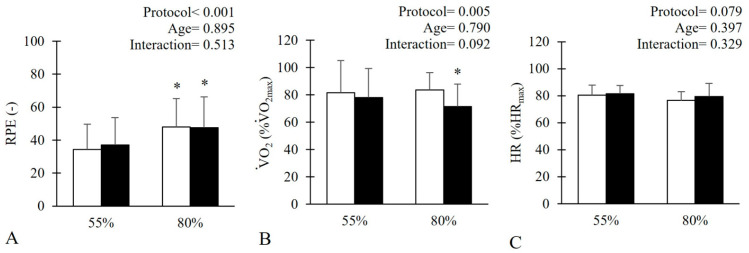
Rating of perceived exertion (RPE, panel (**A**)), relative % oxygen consumption (panel (**B**)), and % heart rate (panel (**C**), i.e., % of the maximum reached during the ramp incremental test, %$\dot{V}$O_2max_, %HRmax) in response to the 55% 1RM and 80% 1RM protocols in young (white) and middle-aged (black) subjects. The main effects of the 2-way RM ANOVA (protocol × age) are displayed in the upper right corner of each panel. * Indicates a significant difference with 55% 1RM for a given group.

Finally, the 80% 1RM protocol was perceived as more difficult to perform by both groups, as confirmed by both a significant main effect of “protocol” in ANOVA and the post hoc analysis. (Figure 4, *p* < 0.001). In relative terms of $\dot{V}$O_2_ and HR, this difference was detectable only in the metabolic response of the middle-aged group, which reported significantly lower %$\dot{V}$O_2_ during the 80% protocol (Figure 4), while no other differences were detected.

## 4. Discussion

This study was aimed at investigating the cardiocirculatory and metabolic responses to training protocols of squat performed with different loads in young and middle-aged individuals. It was hypothesized that middle-aged individuals exhibit similar cardiocirculatory and metabolic responses to RT, providing evidence for the safety of this training modality in this age group. From a cardiocirculatory perspective, we examined similar acute responses in young and middle-aged participants to the training schemes executed respectively with 55% and 80% of the 1RM. The only exception was a higher diastolic blood pressure observed in the middle-aged group, already evident at rest. Likewise, the respiratory and haematological measurements of lactate accumulation demonstrated similar responses in both groups. Notably, the 55% of 1RM elicited a greater metabolic response, attributed to the longer set configuration employed (3 sets of 12 repetitions versus 5 sets of 5 repetitions at 80% 1RM). Overall, this investigation provides the first available data on the “in session” cardiocirculatory safety of RT in middle-aged participants, offering valuable insights for practitioners utilizing this increasingly popular training modality. This, combined with the information on the metabolic responses can assist the design of time-efficient training sessions and contribute to the establishment of best practices.

As determined during the ramp incremental test and the 1RM assessment, the two groups of individuals enrolled in this study presented physical characteristics typical of active, moderately trained subjects (Table 1 [6]), while their blood pressure was “normal” for young and “high normal” for middle-aged subjects (Table 1 [27]).

The training protocols executed with 55% and 80% of the 1RM elicited similar cardiocirculatory responses between young and middle-aged individuals, where no clear difference was detectable in terms of PAs and DP detected between the young and middle-aged participants and between the 55% 1RM and 80% 1RM training protocols (Figure 3). Moreover, although the middle-aged participants showed a higher PAd response to training compared to the young (Figure 3), these values were also present at baseline and remained well within safety limits during exercise [12]. Taken together, these findings support the safety of RT from a cardiocirculatory point of view and suggest that people without hypertension can regulate blood pressure during RT irrespective of increases in external load [13,14].

Similar patterns were recorded between groups on the metabolic responses of $\dot{V}$O_2_, VE, RF, or [La^−^] (Figure 2). However, the middle-aged group displayed a lower metabolic response when performing the 80% 1RM squat protocol in comparison to the 55% 1RM, as indicated by the lower $\dot{V}$O_2_, %$\dot{V}$O_2max_ and, only as a tendency, for [La^−^] (Figure 3 and Figure 4). These findings seem to indicate a reduced activation of both aerobic and anaerobic metabolism in middle-aged participants during shorter, high-load sets (Figure 3) in this ageing group when performing shorter sets with relatively higher loads, and therefore, suggest that longer set configurations could be more effective when RT is aimed at inducing simultaneous musculoskeletal and metabolic adaptations [28]. Although our $\dot{V}$O_2_ data are consistent with the literature for young individuals (i.e., 55% 1RM: 26 ± 7 mL·min^−1^·kg^−1^, 80% 1RM: 24 ± 7 mL·min^−1^·kg^−1^ [14,29]), no other studies have investigated this aspect of training in middle age. This difference may be partly explained by the lower absolute muscle mass typically observed in middle-aged individuals (as supported by the normalized 1RM values, Table 1) and/or plastic changes in muscle composition due to initial ageing (i.e., a progressive shift from type II muscle fibres to type I muscle fibres [30]). Because measures of muscle typology and composition were not performed in this study, further research is warranted to address the interaction of this aspect with the metabolic responses to RT.

Finally, despite the matched training load between the two protocols, both young and middle-aged perceived the 80% 1RM as significantly heavier than the 55% 1RM (Figure 4). This seems to suggest a role of the external load rather than the set duration on the perceived effort of participants, at least when performing back squat with the same recovery intervals. Together with the shorter duration of the 55% 1RM protocol, these results suggest a better time-efficiency of this training configuration for achieving the desired training load, with similar metabolic stimulus (Figure 3 and Figure 4) and comparable cardiocirculatory strain (Figure 2). This has potential implications not only for session structuring but also for cardiovascular safety and long-term adherence, as perceived exertion and session duration are key determinants of training compliance. This may be particularly relevant given the known difficulty in engaging individuals in RT despite strong recommendations to include it as part of a healthy lifestyle [31]. Although these findings are in agreement with other research investigating RPE after squat performed at different % of 1RM [14], practitioners should remain cautious in generalizing them, as they reflect responses to a single exercise. Additionally, RPE may vary considerably within individuals depending on uncontrolled factors, such as sleep quality, prior fatigue, psychological state, or recent nutrition [32], which should be acknowledged when interpreting perceptual responses in applied settings.

### Limitations and Perspectives

It should be acknowledged that the present study has some potential limitations that need to be considered to contextualize the results. First, the sample size was based on previous investigations assessing the $\dot{V}$O_2_ response during aerobic exercise [19] and on initial pilot testing, but this may not directly translateto RT due to the higher signal-to-noise ratio imposed by the intermittent nature of the activity and postural changes. Although we are confident that this issue was at least partially mitigated by the data analysis strategy employed (i.e., mean of peak responses after each set), it should still introduce a degree of uncertainty and be taken into account when planning future research. The same considerations apply to variables that were not primary outcomes of the study (e.g., heart rate; Figure 3), as their variability and effect sizes may differ and therefore require a larger sample to detect meaningful differences. Furthermore, although our sample may be representative of healthy individuals within the general population, it did not include highly (de)conditioned participants and should therefore be extended in future investigations to detect potential differences in physiological responses among individuals of the same age but with very low or very high physical capacity. Finally, it should be considered that the choice to equate global training load translates into different durations of muscular effort across conditions. As a result, transferability to real RT settings increased, but some of the observed physiological differences likely reflected differences in time under tension rather than load alone. From a practical standpoint, this highlights how protocols with similar calculated loads can produce different internal responses depending on how the work is structured. Practitioners should therefore be cautious when using simplified load metrics and consider the influence of volume distribution on training outcomes.

From a broader perspective, our findings support the feasibility and time efficiency of low-load RT (3 × 12 at 55% 1RM), which induced a comparable overall physiological strain but with lower perceived effort than higher-load training. This configuration may facilitate adherence and wider implementation in this population, particularly in light of increasing evidence for the effectiveness of low-load RT [33]. The comprehensive evaluation of cardiovascular, metabolic, and perceptual responses strengthens the translational relevance of the study, while longitudinal studies are warranted to determine whether these acute responses lead to improved adherence, training adaptations, and retention.

## 5. Conclusions

This study investigated the cardiocirculatory and metabolic responses of young and middle-aged individuals performing squat with the lower and higher ranges of the recommended sets and load configurations. While no difference in physiological responses was found in healthy participants belonging to these two age groups, the lower load–higher repetition training scheme appeared to offer better characteristics in terms of time efficiency, metabolic activation, and perceived effort, with the same cardiocirculatory strain. This information may assist practitioners and trainers in developing optimal strategies for RT interventions. Moreover, the reduced perceived effort observed under low-load conditions could support adherence and broader implementation of RT among middle-aged adults, if confirmed by future studies.

## Figures and Tables

**Table 1:** Subjects’ characteristics.

	Young			Middle-Aged			Total			*p*-Value
n°	9			9			18			
Age (yrs)	25.6	±	2.2	50.1	±	5.4	37.8	±	13.2	0.000 *
Weight (kg)	66.7	±	13.0	76.3	±	14.4	71.5	±	14.2	0.160
Height (cm)	1.73	±	0.09	1.74	±	0.07	1.74	±	0.08	0.724
BMI (kg/m^2^)	22.1	±	2.4	24.9	±	3.3	23.5	±	3.2	0.054
$\dot{V}$O_2max_ (mL·kg^−1^·min^−1^)	42.7	±	6.6	34.0	±	6.4	38.4	±	7.7	0.012 *
HR_max (b·min^−1^)	192.3	±	4.4	176.6	±	7.4	184.5	±	10.1	0.000 *
PAs (mmHg)	122.4	±	9.9	131.0	±	8.5	126.7	±	10.0	0.067
PAd (mmHg)	67.8	±	6.8	90.5	±	7.0	79.1	±	13.5	0.000 *
1RM squat (kg)	88.9	±	28.4	78.6	±	25.2	83.7	±	26.6	0.427
1RM norm squat (kg/kg)	1.3	±	0.2	1.0	±	0.3	1.2	±	0.3	0.028 *

Mean ± SD values of anthropometric, physiological and strength variables, in young, middle-aged and the total group. The *p*-value relative to the unpaired *t*-test (young vs. middle-age) is displayed on the right side. * Indicates statistically significant difference between the young and middle-aged groups.

## Data Availability

The data included in this study are available from the corresponding author upon reasonable request.

## Abbreviations

The following abbreviations are used in this manuscript:

RT	Resistance Training
1RM	One Repetition Maximum
$\dot{V}$O_2_	Oxygen Consumption
$\dot{V}$O_2_max	Maximal Oxygen Consumption
PO	Power Output
HR	Heart Rate
$\dot{V}$E	Ventilation
Rf	Respiratory Frequency
[La^−^]	Blood Lactate Concentration
PAs	Systolic Pressure
PAd	Diastolic Pressure
DP	Double Product
RPE	Rating of Perceived Exertion
RM ANOVA	Repeated Measures Analysis of Variance
SD	Standard Deviation
$\dot{V}$O_2_max	Percentage of Maximal Oxygen Consumption
%HRmax	Percentage of Maximal Heart Rate
